# Candidate gene analysis for determinacy in pigeonpea (*Cajanus* spp.)

**DOI:** 10.1007/s00122-014-2406-8

**Published:** 2014-10-21

**Authors:** Reyazul Rouf Mir, Himabindu Kudapa, Sandhya Srikanth, Rachit K. Saxena, Ashutosh Sharma, Sarwar Azam, Kulbhushan Saxena, R. Varma Penmetsa, Rajeev K. Varshney

**Affiliations:** 1 International Crops Research Institute for the Semi-Arid Tropics (ICRISAT), Patancheru, 502 324 Hyderabad India; 2 Division of Plant Breeding and Genetics, Shere-Kashmir University of Agricultural Sciences and Technology of Jammu (SKUAST-J), Chatha, 180 009 Jammu India; 3 Institute of Molecular, Cell, and Systems Biology, College of Medical, Veterinary, and Life Sciences, University of Glasgow, Glasgow, G12 8QQ UK; 4 Department of Plant Pathology, University of California-Davis, Davis, CA 95616 USA; 5 School of Plant Biology and Institute of Agriculture, The University of Western Australia, 35 Stirling Highway, Crawley, WA 6009 Australia

## Abstract

**Key message:**

**We report a likely candidate gene,** ***CcTFL1,*** **for determinacy in pigeonpea through candidate gene sequencing analysis, mapping, QTL analysis together with comparative genomics and expression profiling.**

**Abstract:**

Pigeonpea (*Cajanus cajan*) is the sixth most important legume crop grown on ~5 million hectares globally. Determinacy is an agronomically important trait selected during pigeonpea domestication. In the present study, seven genes related to determinacy/flowering pattern in pigeonpea were isolated through a comparative genomics approach. Single nucleotide polymorphism (SNP) analysis of these candidate genes on 142 pigeonpea lines found a strong association of SNPs with the determinacy trait for three of the genes. Subsequently, QTL analysis highlighted one gene, *CcTFL1*, as a likely candidate for determinacy in pigeonpea since it explained 45–96 % of phenotypic variation for determinacy, 45 % for flowering time and 77 % for plant height. Comparative genomics analysis of *CcTFL1* with the soybean (*Glycine max*) and common bean (*Phaseolus vulgaris*) genomes at the micro-syntenic level further enhanced our confidence in *CcTFL1* as a likely candidate gene. These findings have been validated by expression analysis that showed down regulation of *CcTFL1* in a determinate line in comparison to an indeterminate line. Gene-based markers developed in the present study will allow faster manipulation of the determinacy trait in future breeding programs of pigeonpea and will also help in the development of markers for these traits in other related legume species.

**Electronic supplementary material:**

The online version of this article (doi:10.1007/s00122-014-2406-8) contains supplementary material, which is available to authorized users.

## Introduction

Pigeonpea [*Cajanus cajan* (L.) Millsp.] is one of the most important food legume crops for arid and semi-arid regions of the world. It is grown on ~5 million hectares (ha) globally and constitutes one of the main sources of protein for >1 billion people, as well as a cash crop for millions of resource poor people living in Asia, Africa, South America, Central America and the Caribbean (Mula and Saxena 2010). The pattern and time of flowering are important adaptive traits in flowering plants controlled by physiological signals, genes, gene interactions and interactions of genes with the environment (Liu et al. 2010). Tremendous progress has been made in the area of isolation and characterization of plant genes for crop improvement due to emergence of plant genomics (Arabidopsis Genome Initiative 2000; Mouradov et al. 2002; Michael and Jackson 2013). Availability of genome sequence of a number of plant species together with comparative genomics have helped in answering some of the fundamental aspects of plant biology including identification and analysis of genes involved in adaptive traits in crop species (Cronk 2001; Foucher et al. 2003). One of the best examples of such evolutionary developmental studies in plant species is the identification and analysis of MADS box genes involved in flower development (Ma and De Pamphilis 2000). Subsequently, orthologous genes have been isolated in many species providing insights into the conservation and diversification of such genes and their functions in plant development (Hofer and Ellis 2002).

Several approaches like genetic linkage analysis, candidate gene association analysis, and heterologous transformation have been used to test for the candidacy of homologous genes from *Arabidopsis* into other crop species like soybean (Tian et al. 2010). These studies revealed that flowering time/flowering pattern/determinacy has been selected long ago by breeders in combination with photoperiod insensitivity to obtain varieties with shorter flowering period, earlier maturation and ease of mechanized harvest (Repinski et al. 2012). Genetic mechanism responsible for these traits has been uncovered in model plant *Arabidopsis* (*Arabidopsis* *thaliana*), pea (*Pisum sativum*), soybean (*Glycine max*), common bean (*Phaseolus vulgaris*) etc. (Foucher et al. 2003; Hecht et al. 2005; Kwak et al. 2008; Liu et al. 2010; Repinski et al. 2012). In some cases it was proved that determinacy is controlled by a single gene, whereas in other studies more than one gene was found responsible for the transition of different growth habits (Tian et al. 2010). In pea, it was shown that the determinate mutant (*det*) is caused by mutations in a homologue of the *Arabidopsis* *TFL1* gene (Foucher et al. 2003). In soybean, the gene responsible for determinacy “*GmTfl1*” was isolated and found to complement the functions of *TFL1* in *Arabidopsis* (Liu et al. 2010; Tian et al. 2010). Similarly, in common bean, it was proved that gene “*PvTFL1y*” co-segregated with the determinacy locus “*fin*” (Kwak et al. 2008) and later the same was validated and found as a functional homolog of *Arabidopsis* *TFL1* gene (Repinski et al. 2012). In pigeonpea, both indeterminate (IDT) and determinate (DT) type flowering pattern exist (Mir et al. 2012b). Wild relatives and most of the cultivars have indeterminate growth habit and therefore, it is believed that determinate forms of pigeonpea were selected by farmers or breeders during pigeonpea domestication process or breeding. The availability of determinate growth habit genotypes having initial vigor and tolerance to drought and water logging have been found advantageous over indeterminate types for environments with moderate growth (5–6 t ha^−1^), while as IDT type lines have been found suitable for environments with high (7–8 t ha^−1^) growth potential (Singh and Oswalt 1992). However, only some linked markers associated with flowering pattern/determinacy have been reported recently in pigeonpea (Mir et al. 2012b). The present study reports the isolation of seven genes and identification of likely candidate gene “*CcTFL1*” for determinacy in pigeonpea using candidate gene sequencing, linkage mapping based association analysis, comparative genomics and differential gene expression approaches.

## Materials and methods

### Plant material and phenotyping

A set of 142 pigeonpea germplasm [*Cajanus cajan* (L.) Millsp.] accessions including 84 indeterminate (IDT) and 58 determinate (DT) accessions were selected to test associations of candidate genes/SNPs with determinacy in pigeonpea (Table S1a). For genetic mapping of candidate genes/SNPs, a bi-parental F_2_ mapping population derived from a cross ICPA 2039 (DT, plant height: 140 cm, days to 50 % flowering: 70 to 80 days, days to maturity: 130 to 140 days) × ICPR 2447 (IDT, plant height: 150 cm, days to 50 % flowering: 75 to 85 days, days to maturity: 125 to 135 days) comprising 188 lines was used (Table S1b). To validate the identified SNP in candidate gene “*TFL1*”, another F_2_ mapping population derived from a wide cross [*C. cajan* (ICPL 85010) × *C. volubilis* Blanco (ICP 15774)] comprising of 21 F_2_ lines was used (Table S1c).

Determinacy data were recorded at the Research Farm, ICRISAT, Patancheru, Hyderabad, India in the year 2009 cropping season. For both F_2_ mapping populations, data were recorded on single plants for plant height, flowering time and determinacy in un-replicated manner.

### DNA isolation

Total genomic DNA was extracted from DT/IDT lines, parental lines and segregating F_2_ progenies at an early seedling stage using a high-throughput mini DNA extraction protocol (Cuc et al. 2008). The quality and quantity of extracted DNA was checked on 0.8 % agarose gels and the DNA was normalized to 5 ng/µl for further use.

### RNA isolation

For expression profiling, two pigeonpea accessions ICPA 2039 (DT) and ICPL 87118 or Asha (IDT) were used as representatives of the two phenotypic categories. Seeds were sown in pots (three seeds per pot), and maintained in a glasshouse under controlled conditions. Plants in each pot were thinned to one healthy plant/pot at the stage, 15 days after germination (DAG). Tissues representing different developmental stages viz., root tip, roots, young leaves, mature leaves, shoot, shoot tip and flower were targeted for collection in three biological replications. Six tissue samples (excluding flower, due to limited or no flower) were harvested from individual glass-house grown pigeonpea plants at three different time points, 15DAG, 30DAG, 10 days after flowering (DAF). Seven tissue samples (including flower) were harvested at 20 DAF. Collection of tissues at different growth stages from different parts of the pigeonpea plants (vegetative vs reproductive parts) was based on the evidence that *TFL1* gene shows differential expression in different parts at different stages of plant development in *Arabidopsis* and other related legume crops like pea, soybean and common bean (Repinski et al. 2012). Tissues were washed thoroughly with 0.1 % DEPC water, frozen in liquid nitrogen and stored at −80 °C until RNA extraction. Total RNA was extracted from the harvested tissues using TRIzol (Invitrogen, USA) according to the manufacturer’s protocol. RNA quality was assessed on 1.2 % formaldehyde agarose gels, while purity of RNA was assessed using a NanoVue spectrophotometer (A260/A280 ratio). First strand cDNA was synthesized from total RNA (2.5 μg) using a cDNA synthesis kit (Superscript^®^ III, Invitrogen, CA, USA) following manufacturer’s instructions.

### Selection of candidate genes

A set of seven genes were selected based on the earlier information about their role in determinacy/flowering pattern and photoperiod sensitivity. The details of these genes and their function in *Arabidopsis* are given elsewhere (see Kwak et al. 2008; Table 1).

**Table 1:** List of primer pairs used for amplification of the respective candidate genes

Gene (IDs)	Primer name	Sequence (5′–3′)	Reference
*CcAP1* (Apetela1) (ID: 843244)	AP1-l1-f	AGCTCATGAGATCTCTGTTC	Kwak et al. (2008)
	AP1-l1-r	AGCGYTCIAGHATCTTCTCC	Kwak et al. (2008)
*CcFCA* (Flowering control locus A) **(**EF643224, EF643225, EF643226)	FCA-F1	AAGCAAGCTTTCATTCATCTC	Kwak et al. (2008)
	FCA-R4	GTAACTCCATATGCCTGG	Kwak et al. (2008)
*CcFLD* **(**Flowering locus D) (EF643227, EF643228, EF643229)	FLD-F1	TTGGAATATGCAAATGCTGGG	Kwak et al. (2008)
	FLD-R2	CAGCTTCACCAGCCAC	Kwak et al. (2008)
*CcFKF1* (F-Box1) (EF643231, EF643232, EF643233, EF643234)	FKF1-F1	GTTGTGKCTGAGATTAG	Kwak et al. (2008)
	FKF1-R2	GCTATGWCCCCAAG	Kwak et al. (2008)
CcGI (Gigantea) (EF643235, EF643236, EF643237, EF643238)	GI-R4	CATTTGAGCTGTAACTCCAAG	Kwak et al. (2008)
	GI-F3	GAGAATTTGCACCATTTGGG	Kwak et al. (2008)
*CcTFL2* (Terminal Flower 2) (NC_003076)	TFL2-F	TTCTGTCAAGAGGTTCAAGAG	Kwak et al. (2008)
	TFL2-R	TCCACCATCACTTCTGTTCC	Kwak et al. (2008)
*CcTFL1* **(**Terminal Flower 1) (EF643247, EF643248, EF643249, EF643250)	TFL1-3	GATGTTCCWGGWCCTAGTGAYCC	Kwak et al. (2008)
	CcTFL1_R_Glyma	GCATACACACGGGTCAAACTAGAA	Present study
	CcTFL1_f5b_F	GCCTCTAATAGTGGGAAGAGTC	Present study
	CcTFL1_f5a_R	TTGATGTGATGAAAGGATGC	Present study
	CcTFL1_f6a_F	ACCACATAGCCACTGGATTC	Present study
	CcTFL1_f6a_R	ACATGTGAGGATCAATTTCG	Present study
Allele specific primers for the gene *CcTFL1*			
	TFL1_PCR_CF	GGTACTCATTATACCATCATTTGAG	Present study
	TFL1_PCR_CR	GCATTGAAGTAGACAGCAGC	Present study
	TFL1_PCR_A	GGATTCTTTTAACAACTCAACAAAAA	Present study
	TFL1_PCR_T	GTACTTTTAAATGATTATCTTAAAAA	Present study
qRT-PCR primers for the gene *CcTFL1*			
	CcTFL1_e1_F	GAGCCTCTAATAGTGGGAAGAG	Present study
	CcTFL1_e1_R	TCACCACCATCAATCTCAAC	Present study
	CcTFL1_e2 + 3_F	GTCAACACCATACCCAAGGT	Present study
	CcTFL1_e2 + 3_R	TGTTGTGCCTGGAATATCTG	Present study
	CcTFL1_e4_F	GGATCCATAGGTTTGTGTTTG	Present study
	CcTFL1_e4_R	CCCTCTGTGCATTGAAGTAG	Present study

### Polymerase chain reaction (PCR) and amplicon sequencing

The PCR master mix components and PCR cycle profile used were as described for candidate gene amplification/sequencing in chickpea (Gujaria et al. 2011). PCR products were separated on 1.2 % agarose gels.

PCR products were treated with exonuclease I (Exo) and shrimp alkaline phosphatase (SAP) before subjected to Sanger sequencing from both ends using respective forward and reverse primers at Macrogen Inc., Seoul, South Korea (http://www.macrogen.com/).

### Sequence diversity estimation

Sequencing data were inspected manually for possible sequencing error and consensus sequences were prepared using DNA Baser v 2.9 software (http://dnabaser.com). Consensus sequences for all genotypes were aligned using Clustal W (http://www.ebi.ac.uk/Tools/clustalw2/index.html) (Thompson et al. 1994) and analyzed in BioEdit version 7.0.5.3. for SNP identification.

FASTA multiple sequence alignment files (were analyzed using the SNP DIVersity ESTimator (DIVEST) software module (http://hpc.icrisat.cgiar.org/Pise/5.a/statistics_calculation/ *)* developed at ICRISAT (Jayashree et al. 2009) for calculating the polymorphism information content (PIC) value of individual SNPs as well as nucleotide diversity (*π*), number and PIC value of haplotypes for each gene.

### Genotyping assays

#### CAPS assay

In cleaved amplified polymorphic sequences (CAPS) assay (Konieczny and Ausube 1993) PCR amplicons were subjected to restriction enzyme digestion followed by electrophoretic separation on agarose gels (3 % agarose, 1X TBE buffer, 1 h, 120 V) and visualized by means of ethidium bromide staining (Varshney et al. 2007).

#### dCAPS assay

In derived cleaved amplified polymorphic sequences (dCAPS) assay, sequences on each side of a SNP were provided to the dCAPS Finder 2.0 program (http://helix.wustl.edu/dcaps/) for dCAPS primer design and identification of restriction enzymes for genotyping (Neff et al. 2002).

#### Allele-specific marker assay

Primers targeting each allele of the SNP in gene *CcTFL1* and one pair of external primers were designed using the software tools Fast PCR (Kalendar et al. 2009) and Primer 3 (http://frodo.wi.mit.edu/). Primers were multiplexed into a single PCR reaction to obtain co-dominant marker. This marker assay consisted of two external common primers (external common forward primer- TFL1_PCR_CF and external common reverse primer- TFL1_PCR_CR) flanking the SNP and one internal primer targeting one SNP allele “A-allele” (TFL1_PCR_A) and the other internal primer targeting the other SNP allele “T-allele” (TFL1_PCR_T).

### Genetic mapping and linkage analysis

Genotyping data generated from 188 F_2_ plants derived from cross ICPA 2039 × ICPR 2447 were combined with the data for 81 SSR markers already available on the same population “ICPA 2039 × ICPR 2447” (Bohra et al. 2012). Markers were tested for linkage using JoinMap^®^ 4 program (Ooijen 2006); http://www.kyazma.nl) using LOD 3-10 and the Kosambi map function. The inter-marker distances calculated from the JoinMap^®^ 4 program were used to construct a linkage map which was displayed using MAPCHART version 2.2 (Voorrips 2002).

Single marker regression analysis was carried out in Excel 2007 (Microsoft) using the F_2_ marker genotypes as independent variables and the F_2_ -phenotypes as dependent variables. The phenotypic data were recorded on single F_2_ plants. Composite interval mapping (CIM) (Zeng 1993, 1994) was conducted using WinQTL Cartographer, version 2.5 (for more details see Ravi et al. 2011; Mir et al. 2012b).

### Comparative gene analysis

BLASTN analysis of *CcTFL1* gene of pigeonpea was conducted against the genome sequences of common bean and soybean available at the Phytozyme database (http://www.phytozome.net/). After identification of collinear regions encompassing *TFL1* orthologous in pigeonpea (chromosome 3), soybean (chromosome 19) and common bean (chromosome 1) syntenic relationships were analyzed using SyMAP 4.0 (Soderlund et al. 2011).

### qRT-PCR assay for validation of *CcTFL1* for determinacy

Quantitative real-time PCR (qRT-PCR) was performed using an Applied Biosystems 7500 Real-Time PCR machine and SYBR green chemistry according to the manufacturer’s instructions (Applied Biosystems, CA, USA). Gene-specific primers for qRT-PCR were designed using Primer Express software (Applied Biosystems, CA, USA). Three primer pairs were designed covering all four exonic regions of the *CcTFL1* gene; one primer pair each for exons 1 and 4, one primer pair covering exons 2 and 3. Transcript levels were normalized to glyceraldehyde 3-phosphate dehydrogenase (GAPDH) and β-actin reference genes. PCR was carried out as described in Rawat et al. (2012) and relative expression levels were determined using the 2^−ΔΔCT^ method and student’s *t* test was used to calculate significance (Livak and Schmittgen 2001).

## Results

### Flowering related genes

Seven genes were selected as potentially important agronomic markers based on previous information on their roles in determinacy/flowering pattern/photoperiod sensitivity in *Arabidopsis*, soybean and common bean (Kwak et al. 2008; Tian et al. 2010). A total of 68 primers including 7 degenerate and 61 nested primers were used for amplification of pigeonpea homologues of these genes (Table 1; Table S2). BLASTN analysis showed that the amplified partial gene sequences were most similar to soybean and common bean genes (Table 2). For the *TFL1* gene, maximum similarity at the nucleotide level (80 % identity) was found with the *Dt1* genes of soybean (Table 2). Similarity of *TFL1* sequence at translated protein level was 93 to 95 % with the *Dt1* (soybean), *TFL1a* (pea) and *TFL1y* (common bean). Further efforts were made towards isolation of full-length *TFL1* gene using the whole-genome sequence of pigeonpea (Varshney et al. 2012) and a full-length gene (~1,326 bp) having four exons and three introns was isolated from the CcLG03 of draft genome assembly. This full-length pigeonpea gene sequence showed two most significant hits with soybean (SoyBase.org), one on LG03 with Glyma03g35250.1 and another on LG19 with Glyma19g37890.1; a closest paralogous gene of Glyma03g35250.1 in soybean. The corresponding region on LG19 was recently shown to be the expected soybean gene *GmTFL1* (Glyma19g37890.1) responsible for indeterminacy (Li et al. 2013; Tian et al. 2010). Nucleotide similarity analysis between pigeonpea *TFL1* and Glyma19g37890.1 revealed 81 % identity between the two genes with 11 % gaps. To confirm whether the gene structure of *TFL1* of pigeonpea is similar to that of *GmTFL1* of soybean, we compared the amino acid sequence of the two and found that these two possess the similar protein sequence with 94 % identity.

**Table 2:** BLASTN similarity between pigeonpea amplicons corresponding genes in soybean, common bean and *Arabidopsis*

Gene	Description	*E*-value	Max. identity (%)
*CcAP1*	PREDICTED: floral homeotic protein APETALA 1-like [*Glycine max*]	1e−33	96
*CcFCA*	PREDICTED: *Glycine max* flowering time control protein FCA-like, mRNA	2e−68	92
*CcFLD*	*Phaseolus vulgaris* cultivar Midas flowering locus D (FLD) gene, partial cds	8e−152	92
*CcFKF1*	*Glycine max* circadian clock-associated FKF1 (FKF1), mRNA > gb|DQ371902.1| Glycine max circadian clock-associated FKF1 (FKF1) mRNA, complete cds	0.0	91
*CcGI*	PREDICTED: *Glycine max* protein GIGANTEA-like, transcript variant 2 (LOC100779044), mRNA	0.0	93
*CcTFL2*	*Arabidopsis thaliana* TFL2 gene for TERMINAL FLOWER 2, complete cds	0.004	74
*CcTFL1*	*Glycine max* cultivar Heimoshidou *Dt1* gene	0.00	81

### Sequence diversity

Analysis of amplicon sequence data using the DIVEST program provided a total of 276 SNPs in 6,741 bp sequence data generated for 109 to 142 accessions for 7 genes. The number of SNPs varied from 6 in gene *CcFLD* (SNP frequency = 1/80 bp) to 65 SNPs in gene *CcTFL1* (with a frequency of 1/20 bp). The nucleotide diversity index (*π*) ranged from 2.3 × 10^−3^ (in gene *CcFLD*) to 11.1 × 10^−3^ (in gene *CcGI*) with a mean of 5.4 × 10^−3^ (see Table 3). The polymorphism information content (PIC) values of SNPs varied from 0.03 to 0.16 (average 0.08). Sequence data for these gene regions were analyzed in terms of haplotypes as well. Number of haplotypes observed varied from 1 (in gene *CcGI* and *CcTFL2*) to 20 (in gene *CcTFL1*) with an average 7.42 haplotypes per gene. Haplotype diversity estimated was higher for genes *CcGI* (1.009) as compared to other genes, with lowest for gene *CcFLD* (0.194). While analyzing the sequence data within groups of DT *vs* IDT lines, a higher level of sequence diversity in terms of number of SNPs, SNP frequency, nucleotide diversity and number of haplotypes was noticed in IDT group for most of the genes than in DT group (Table 3).

**Table 3:** Diversity features for the candidate genes in a set of 142 *Cajanus* lines

Gene	No. of genotypes surveyed	Sequence data surveyed (average bp)	No. of SNPs identified	SNP frequency	Nucleotide diversity	PIC of individual SNP	No. of haplotypes	Haplotype diversity	PIC of haplotypes
*CcAP1*									
Determinate	48	1,175	9	1/130.56	0.0008	0.12	7	0.818	0.801
Indeterminate	70	1,175	29	1/40.52	0.0049	0.07	7	0.289	0.285
Across all	119	1,175	37	1/31.76	0.0045	0.05	13	0.624	0.619
*CcFCA*									
Determinate	43	971	14	1/69.36	0.0026	0.09	2	1.009	0.986
Indeterminate	74	971	23	1/42.22	0.0042	0.05	14	0.490	0.483
Across all	117	971	37	1/26.24	0.0039	0.076	2	1.007	0.998
*CcFLD*									
Determinate	48	480	0	0	0	0	1	0	0
Indeterminate	70	480	6	1/80.00	0.0026	0.05	6	0.164	0.162
Across all	118	480	6	1/80.00	0.0023	0.03	6	0.194	0.192
*CcFKF1*									
Determinate	58	1,350	8	1/168.75	0.0010	0.10	2	1.02	0.998
Indeterminate	84	1,350	36	1/37.50	0.0035	0.08	14	0.485	0.48
Across all	142	1,350	42	1/32.14	0.0037	0.06	2	1.007	1
*CcGI*									
Determinate	47	771	25	1/30.84	0.0073	0.19	1	1.022	1
Indeterminate	62	771	21	1/36.71	0.0058	0.10	13	0.637	0.627
Across all	109	771	45	1/17.13	0.0111	0.16	1	1.009	1
*CcTFL2*									
Determinate	53	676	25	1/27.04	0.0055	0.12	1	1.019	1
Indeterminate	73	676	22	1/30.73	0.0064	0.10	9	0.485	0.478
Across all	126	676	44	1/15.36	0.0093	0.08	1	1.008	1
*CcTFL1*									
Determinate	56	1,318	35	1/37.65	0.0014	0.17	12	1.013	0.995
Indeterminate	81	1,318	56	1/23.53	0.00 23	0.13	13	1.01	0.998
Across all	137	1,318	65	1/20.27	0.00 31	0.10	20	1.006	0.999

### Association between candidate genes and determinacy

**Fig. 1:**
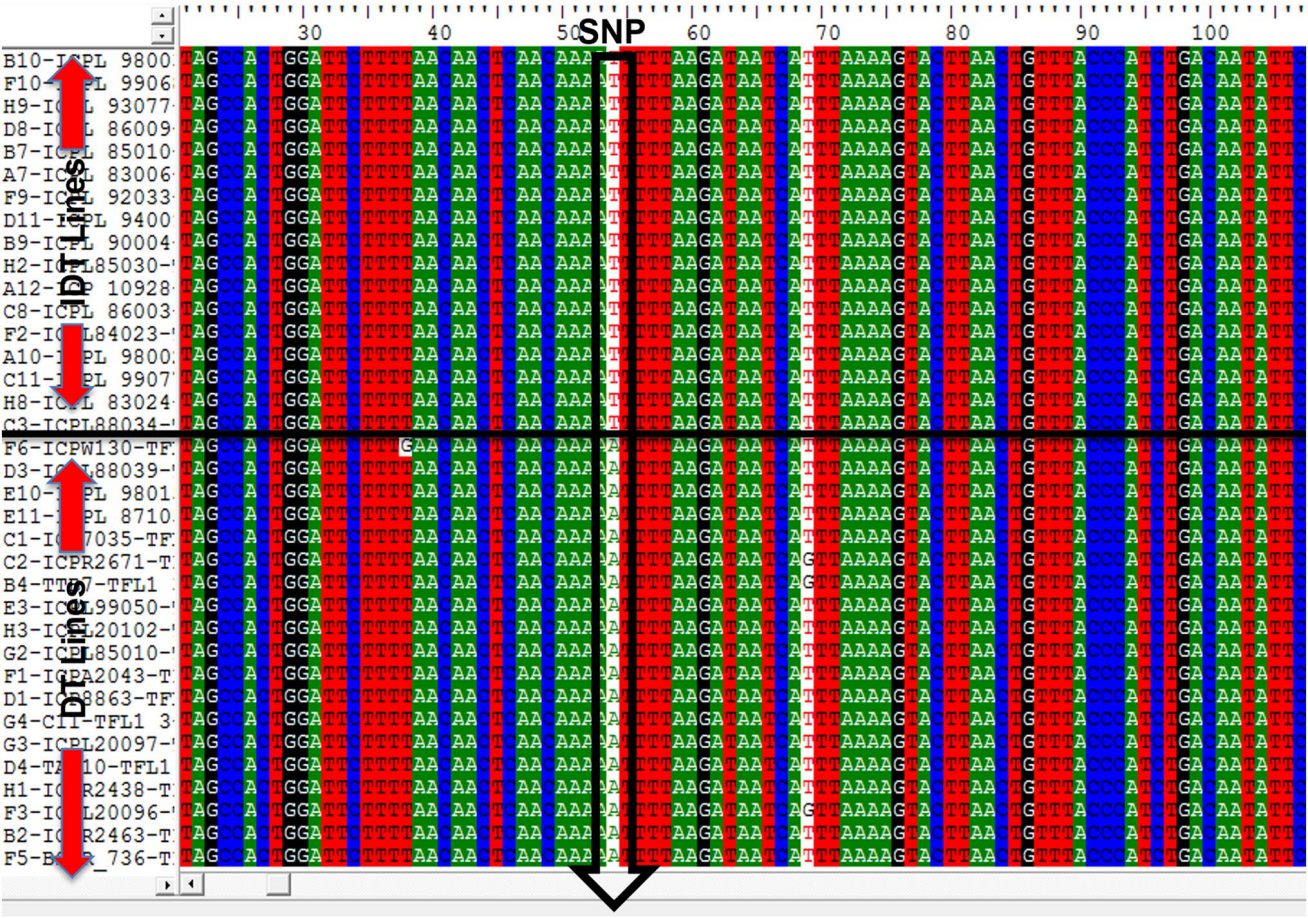
Identification of candidate SNP (A/T) in *CcTFL1* gene showing significant association with determinacy in pigeonpea. The *figure *shows the aligned sequences of IDT and DT germplasm lines. SNP allele “T” is present in all the DT lines and allele “A” is present in all the IDT lines

**Fig. 2:**
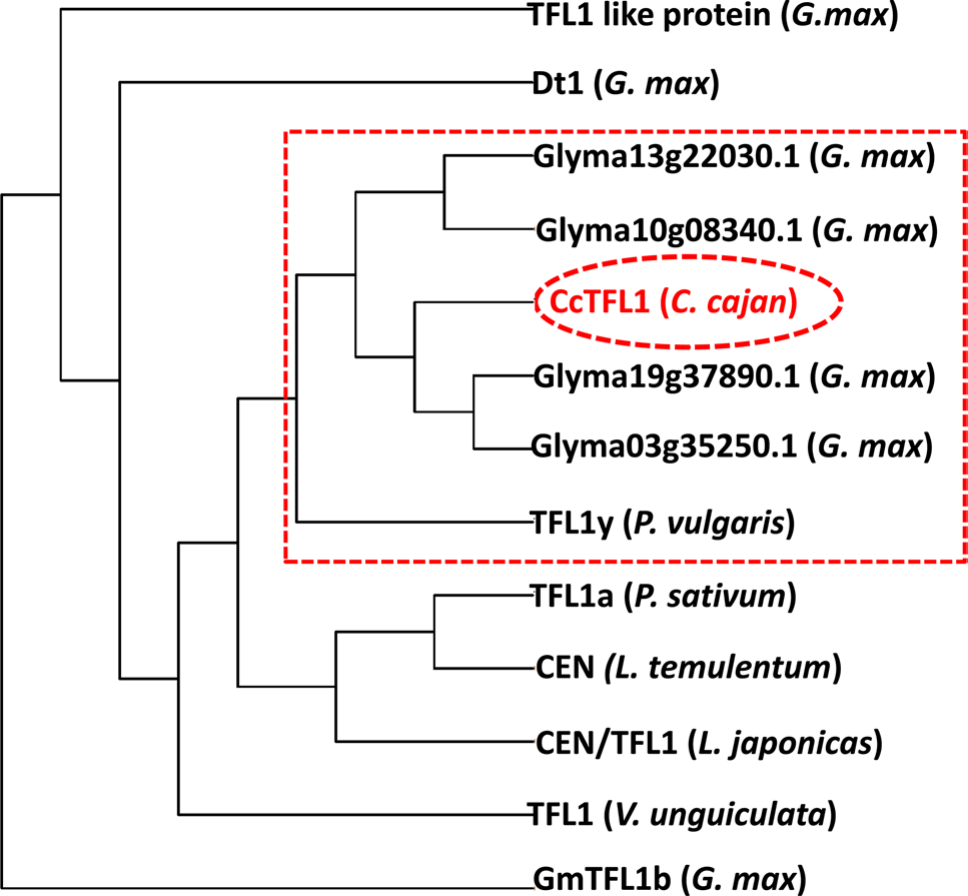
Comparison of pigeonpea Cc*TFL1* with *TFL1* genes in different crops. The *figure* shows that *CcTFL1* clustered with genes for determinacy in *G. max* and *P. vulgaris*

**Fig. 3:**
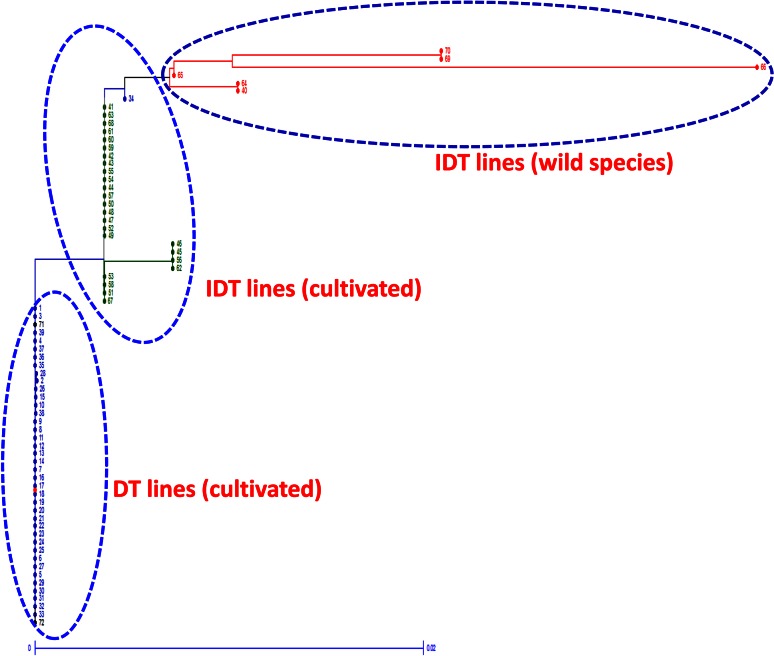
Phylogenetic analysis of pigeonpea DT/IDT lines and wild species using *CcTFL1.* The *figure* shows distinct clustering pattern shown by *CcTFL1*. The DT lines were clearly discriminated from the IDT lines including wild species

In order to test for associations of SNPs with determinacy, all accessions were assigned to one of two phenotypic categories: determinate (DT) or indeterminate (IDT). Three SNPs, one each in gene *CcAP*, *CcGI* and *CcTFL1*, showed strong association with determinacy or indeterminacy. The “A” allele of SNP (A/G) in gene *CcAP1* was present in 100 % (71/71) IDT lines, while the other allele (“G”) was present in 66 % (32/47) DT lines. In the case of *CcGI* gene, the “C” allele was present in ~61 % (29/47) of DT lines while the “A” allele was present in ~61 % (38/62) of IDT lines. The “T” allele of the diagnostic SNP in gene *CcTFL1* discriminated all DT lines (58) from IDT lines (84) with “A” allele with exception in four lines (Fig. 1). These results suggested that genes *CcAP1* and *CcTFL1* could be candidate genes for the determinacy trait in pigeonpea. However, among the three promising genes, *CcTFL1* was considered likely candidate since it could discriminate 100 % DT lines from the IDT lines except four IDT lines which possessed DT alleles. These findings were also supported by the sequence comparison of pigeonpea *CcTFL1* with *TFL1* of the other plants, wherein the clustering pattern revealed maximum similarity of *CcTFL1* with the soybean *GmTFL1* gene models and common bean *PvTFL1y* (Fig. 2). Similarly, *CcTFL1* gene was found useful in phylogenetic classification/analysis of DT and IDT lines including wild pigeonpea accessions (Fig. 3) again indicating its candidacy for determinacy trait in pigeonpea.

### Linkage analysis of candidate genes

**Fig. 4:**
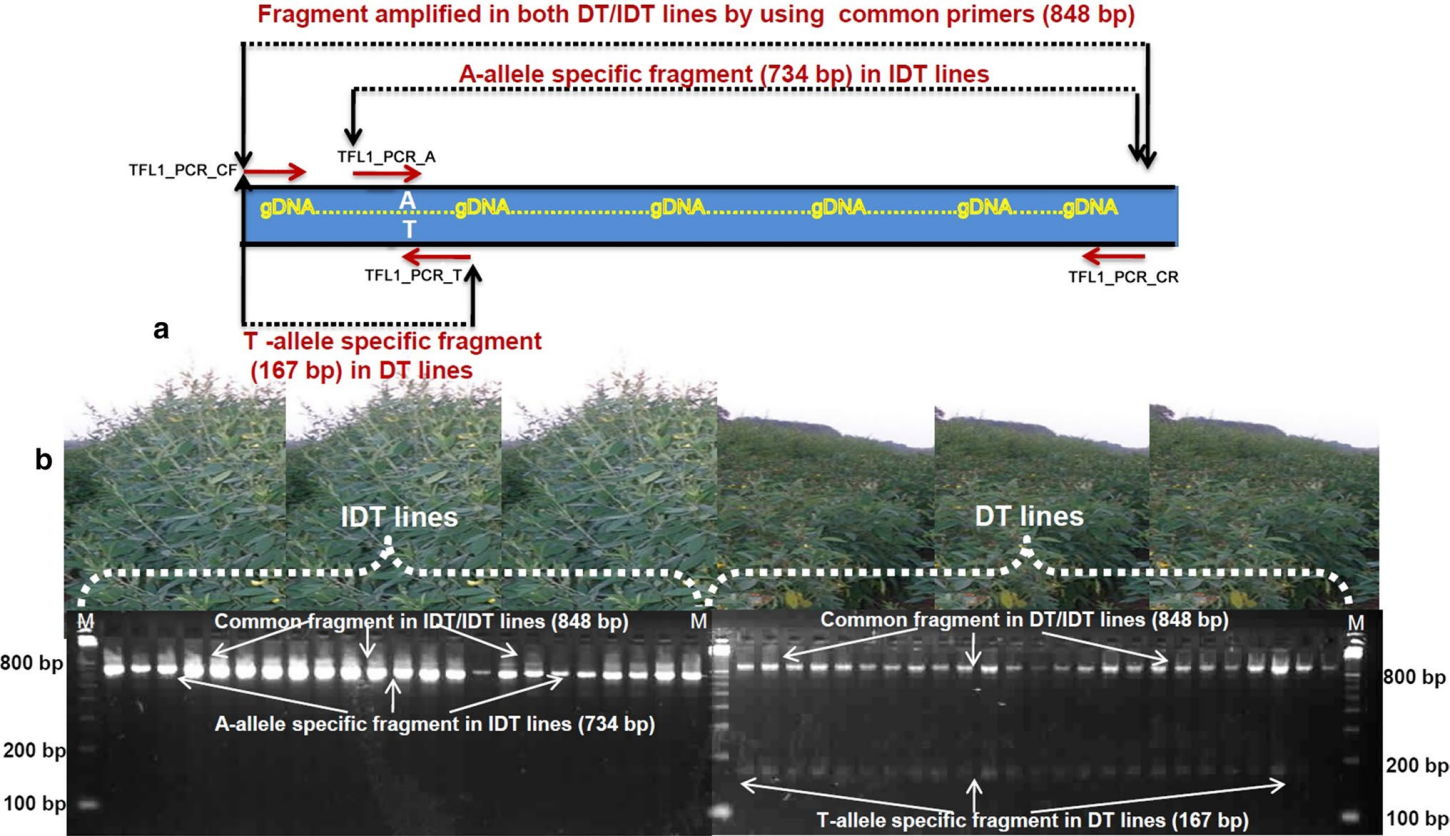
Strategy used for designing allele-specific marker assay for A/T SNP in gene *TFL1* and its validation on DT and IDT lines. **a** Primer designing for allele-specific amplification: from the gene sequence, one pair of external primers including one common forward primer **(**TFL_PCR**_**CF) and one common reverse primer (TFL_PCR_CR) and allele-specific primers (one for “A”- allele specific primer called TFL1_PCR_A (734 bp) and one for “T” allele-specific primer called TFL1_PCR_T (167 bp) were designed, **b** amplification pattern of allele-specific marker assay developed for the SNP (A/T) in gene *CcTFL1*: by using above mentioned primer pairs, DNAs of IDT and DT lines showed amplification of “A” allele (734 bp) in all IDT lines and “T” allele (167 bp) in all DT lines. Common fragment (848 bp) was amplified in both IDT and DT lines

To determine the candidate gene(s) out of three promising genes, we followed linkage analysis approach in which attempts were made to map the promising genes and test their linkage with the determinacy trait in pigeonpea. CAPS and dCAPS assays for linkage analysis were based on the SNPs in genes *CcAP1* and *CcGI*, respectively. A co-dominant, allele-specific marker assay was developed for the SNP (A/T) in the gene *CcTFL1* whereby an 848-bp amplicon is present in both DT and IDT lines, a 734-bp amplicon is specific to IDT lines, and a 167-bp amplicon is specific to DT lines (Fig. 4a, b).

The markers were used to score 188 lines of the F_2_ mapping population derived from ICPA 2039 (DT) × ICPR 2447 (IDT). The phenotypic evaluation of 188 F_2_ progenies for DT/IDT growth habit revealed that 152 progenies possessed IDT growth habit whereas 36 progenies possessed DT growth habit. The genotyping of this population with gene *CcGI* showed DT-specific fragment in 66 F_2_ lines and IDT-specific fragment in 15 F_2_ lines whereas majority of lines (106) showed heterozygous nature with only one line with missing data. Similarly, the genotyping of *CcTFL1* on 188 F_2_ lines of bi-parental mapping population showed segregation for DT/IDT. For instance, out of 36 DT progenies, 26 showed DT fragment, 5 showed both DT and IDT fragments (heterozygous), 2 showed IDT fragment and remaining 3 showed failure in amplification (missing data). Likewise, out of 152 IDT progenies, 71 lines showed IDT fragment and 80 lines showed both DT and IDT fragment (heterozygotes) whereas one progeny showed failure in amplification (missing data).

**Fig. 5:**
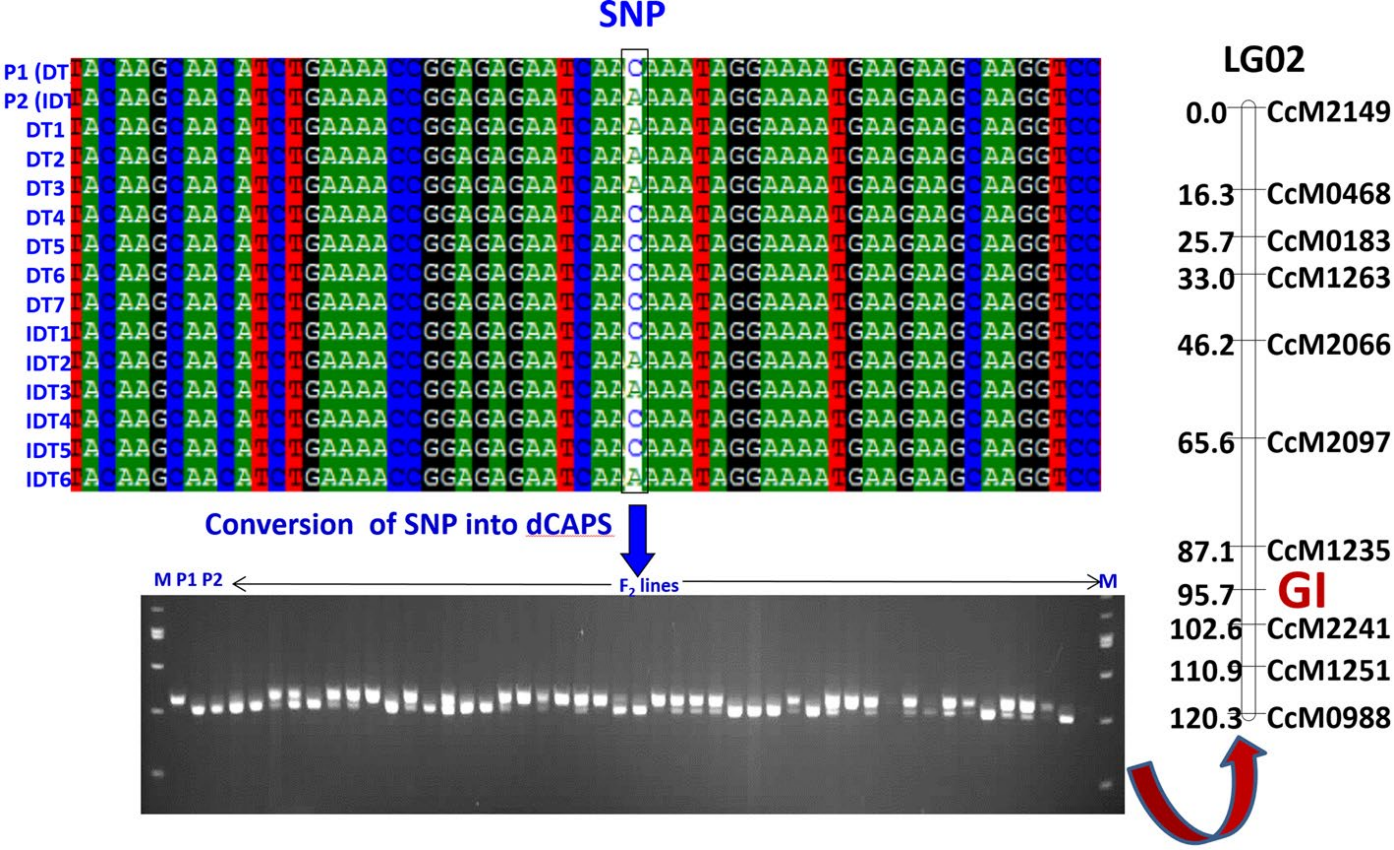
Genetic mapping of candidate gene *Gigantea* (*GI*) on LG02 using F_2_ mapping population derived from ICPA 2039 × ICPR 2447. The *figure* shows identification and polymorphism by SNP (A/C) between the two parental genotypes and the F_2_ lines of mapping population and its conversion into dCAPS marker assay for genotyping and genetic mapping

**Fig. 6:**
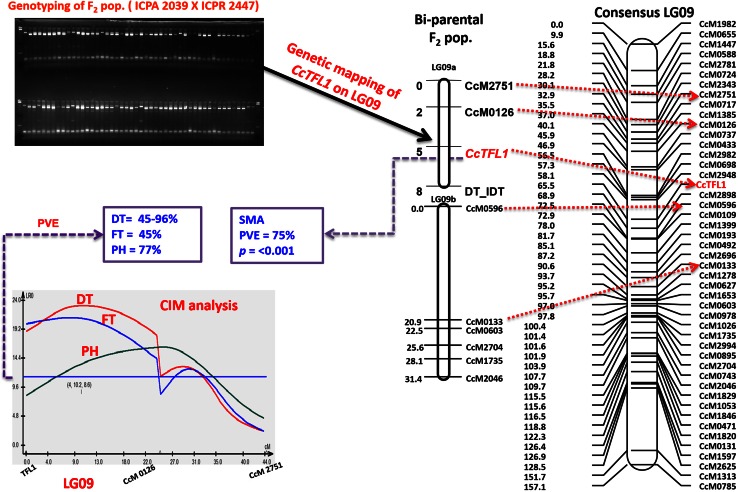
Genetic mapping and linkage analysis of Cc*TFL1* for determinacy, flowering time and plant height. The *figure* shows genotyping of F_2_ mapping population using allele-specific marker assay for *CcTFL1* followed by its mapping on the LG09 of bi-parental mapping population and on the consensus map and QTL analysis (CIM). Important genomic region shown on LG09 harbors QTLs for determinacy, flowering time and plant height in the marker interval defined by *CcTFL1* and CcM0126

The genotyping data generated were used in conjunction with existing genotyping data for 81 SSR markers (Bohra et al. 2012). As a result, *CcGI* and *CcTFL1* were mapped. The gene *CcGI* mapped to LG02 in the vicinity of SSR markers *CcM1235* and *CcM2241* (Fig. 5), while gene *CcTFL1* was linked to marker CcM0126 on LG09 of individual genetic map of ICPA 2039 × ICPR 2447 as well as the consensus map of pigeonpea (Fig. 6) (Bohra et al. 2012). No linkage was detected with marker CcAP1.

Single marker analysis (SMA) using regression and composite interval mapping (CIM) based on our genotype and phenotype data showed association of *CcTFL1* with determinacy as well as flowering time and plant height. For instance, SMA analysis of *CcTFL1* with trait determinacy showed gene-trait association explaining 75 % phenotypic variation. On the other hand, CIM analysis revealed a cluster of three major QTLs one each for determinacy, flowering time and plant height present in the genomic region (24 cM) defined by *CcTFL1* and CcM0126 (Fig. 6). This genomic region explains 45–96 % phenotypic variation for determinacy, 45 % for flowering time and 77 % for plant height (Fig. 6).

**Fig. 7:**
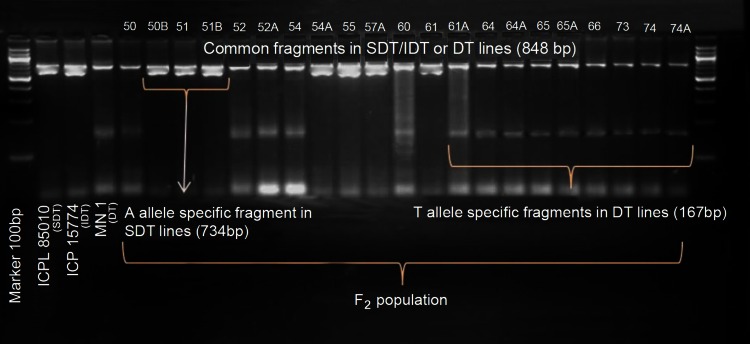
Validation of IDT- or DT-specific alleles for *TFL1* gene in F_2_ progenies of the cross *C. cajan* (ICPL 85010) × *C. volubilis* (ICP 15774). The *figure* shows amplification of common fragment (848 bp) and “T” allele-specific fragment (167 bp) in DT lines including the parental (check) genotype ‘MN1’, while SDT lines including parental lines, ICPL 85010 and ICP 15774 showed amplification of common fragment (848 bp) and “A” allele-specific fragment (734 bp)

The results of linkage analysis revealed gene *CcTFL1* as the most promising gene among the three (*CcAP1*, *CcGI* and *CcTFL1*) genes for determinacy in pigeonpea. Further to validate the association of *CcTFL1* with determinacy trait in pigeonpea, another mapping population segregating for determinacy and semi-determinacy derived from a wide cross [*C. cajan* (ICPL 85010) × *C. volubilis* (ICP 15774)] comprising of 21 F_2_ lines was used. Out of these two parents, cv. *C. cajan* was a semi-determinate (SDT) and wild *C. volubilis* was an IDT line. The F_2_ individuals segregated for DT (14 plants) and SDT (7 plants) growth habits. Less number of F_2_ individuals is due to development of only one F_1_ plant in above wide cross. In earlier reports for growth habit inheritance in pigeonpea, SDT growth habit was found as a result of separate gene in SDT × DT crosses. However, in SDT × IDT crosses, IDT showed epistatic behavior over SDT in F_1_ and while in F_2_ all the patterns like IDT, SDT and DT were observed (see Gupta and Kapoor 1991; Gumber and Singh 1997). The type of segregation in our cross between an SDT and IDT lines (with F_1_ being SDT) is possible in case IDT parent is in heterozygous condition and the SDT parent may be either in homo- or in heterozygous condition. The variation in expected segregation ratio may be as a result of mutations in wide crosses. Nevertheless, more detailed analysis for this segregation needs to be worked out by developing more F_1_/F_2_s separately. Allele-specific marker assay developed for the SNP (A/T) was used to genotype 21 F_2_ progenies. Two fragments including one common fragment (848 bp for both DT and IDT) and one allele-specific fragment (734 bp- IDT-specific/167 bp- DT-specific) were observed in F_2_ progenies tested. The degenerated common primers (TFL1_PCR_CF and TFL1_PCR_CR) amplified 848 bp-specific fragment among all genotypes. The IDT and SDT genotypes exhibited “A” allele-specific fragment (734 bp), whereas DT genotypes exhibited “T” allele-specific fragment (167 bp). This marker clearly distinguishes IDT lines from the DT lines based on amplification of specific fragments in IDT lines (734 bp) and DT lines (167 bp) in addition to amplification of common fragment in both DT and IDT lines (848 bp fragment). Among F_2_ progenies, all DT plants (50, 52, 52A, 54, 60, 61A, 64, 64A, 65, 65A, 66, 73, 74 and 74A) showed DT-specific fragment (167 bp) and the common fragment (848 bp), while the remaining F_2_ plants that were SDT (50B, 51, 51B, 54A, 55, 57A and 61) showed IDT-specific fragment (734 bp) and the common fragment (848 bp) (Fig. 7). These results validated association of *CcTFL1* with determinacy trait in pigeonpea.

Further, the likely candidature of *CcTFL1* for determinacy through the first approach, linkage analysis was validated through two more approaches- comparative mapping and expression profiling using qRT-PCR. The second approach (comparative mapping) was followed for the gene *CcTFL1* to compare its syntenic relationship with the genomic regions harboring determinacy gene in soybean and common bean. In the third (expression analysis using qRT-PCR) approach, functional validation to confirm candidacy of *CcTFL1* for determinacy in pigeonpea was conducted.

### Comparative genomics analysis

**Fig. 8:**
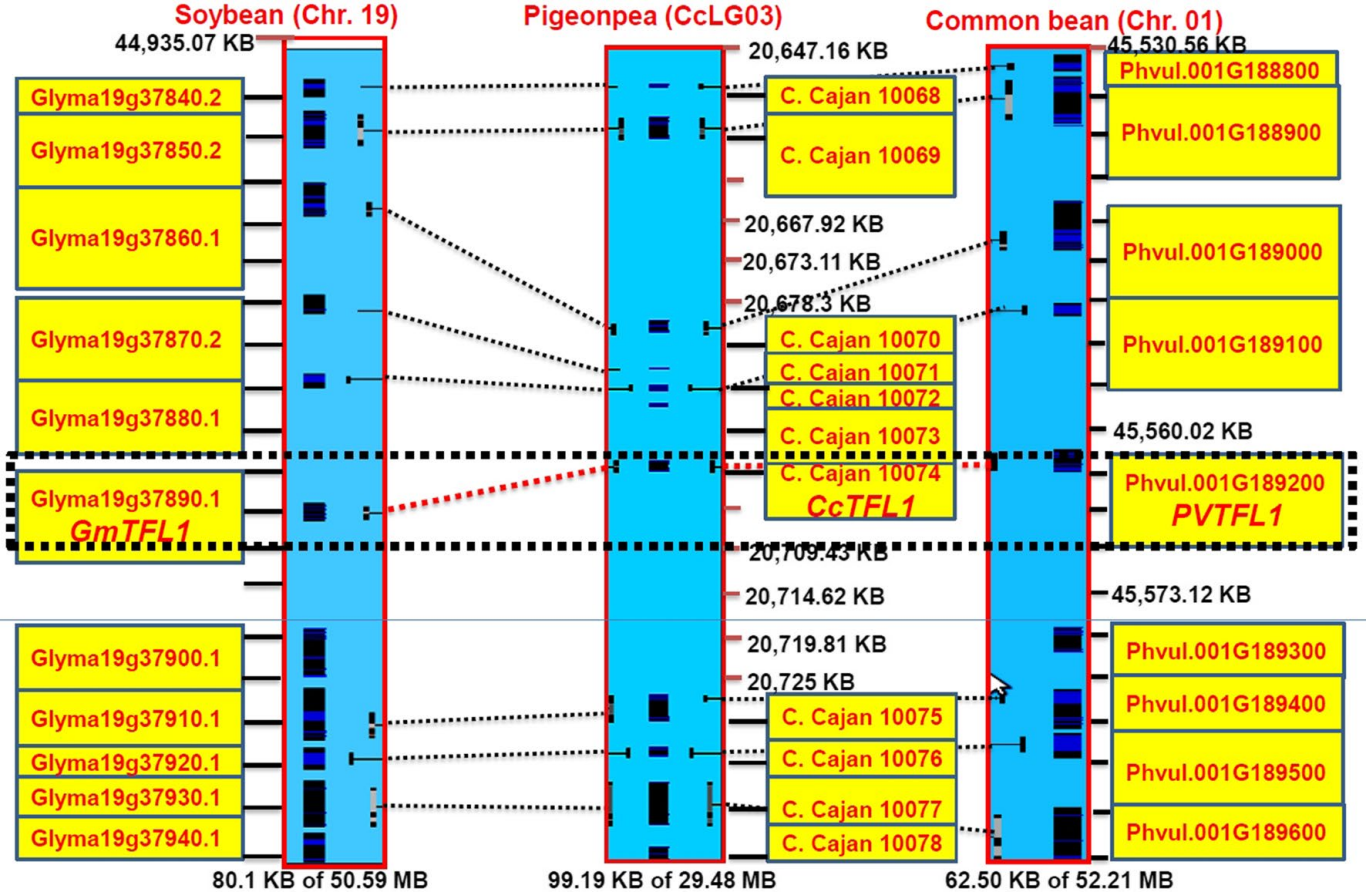
Synteny of pigeonpea genomic region containing *CcTFL1* with the corresponding soybean and common bean genomic regions. The *figure* shows synteny between the three legume genomes at genomic region containing *CcTFL1*

As determinacy is an important trait in other legume species like soybean and common bean of the *Phaseoloid* clade, genome sequences of soybean and common bean were analyzed for the *CcTFL1* gene sequence. Stringent BLASTN analysis provided a single prominent hit on chromosome 1 (45,562,544–45,561,745 bp) of common bean (79.30 % sequence identity, *E*-value 0.0) and on chromosome 19 (44,980,787–44,979,944 bp) of soybean (78.58 % sequence identity, *E*-value 0.0). With an objective to understand gene conservation at a micro-syntenic level, a 50-kb region (20,647–20,747 kb) of the pigeonpea genome flanking *CcTFL1* was aligned with the corresponding syntenic regions in soybean (Chr. 19, 44,938–45,011 kb) and common bean (Chr.1, 45,530–45,593 kb). Detailed analysis showed conservation of eight gene sequences in this region across the three legume crops (Fig. 8). This high-level of conservation of gene sequence in homologous region across three *Phaseoloid* legumes confirmed the orthologous nature of *CcTFL1* gene. This analysis, therefore, enhanced confidence further in assuming *CcTFL1* gene as a candidate gene for determinacy in pigeonpea.

### Expression analysis of *CcTFL1*

**Fig. 9:**
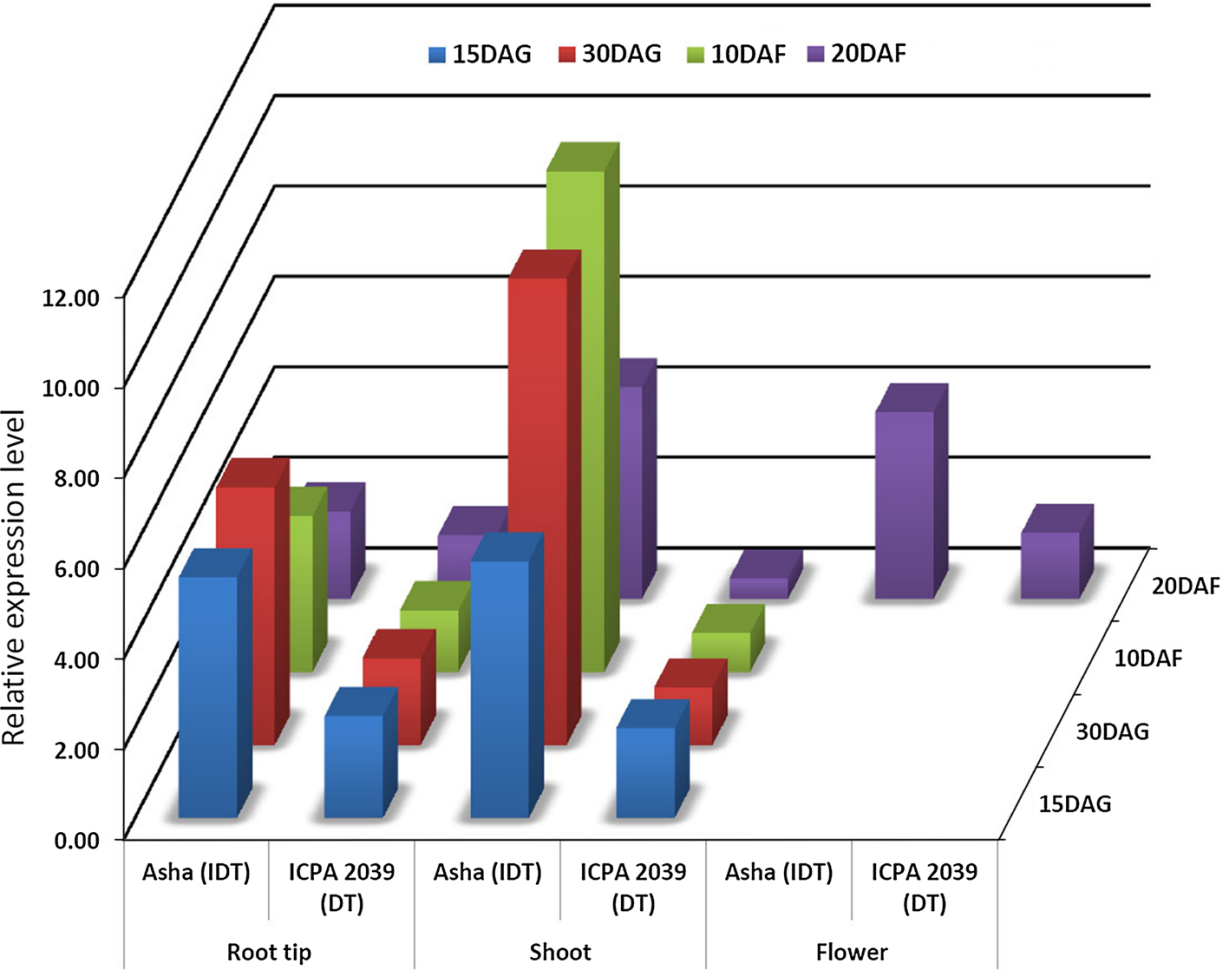
Differential expression profiles of gene *CcTFL1* for determinacy in pigeonpea. The *figure* shows down regulation of gene at different stages of plant growth viz, root tips, shoot, flowers of DT line ICPA 2039 when compared to the IDT line Asha (ICPL 87119)

With a final objective to corroborate *CcTFL1* as a determinacy gene, qRT-PCR analysis was performed on tissues from a representative IDT and DT accessions, cultivar Asha (ICPL 87119- IDT) and line ICPA 2039 (DT). In root tips, shoots and flowers, expression levels of the candidate *CcTFL1* were consistently lower (by 2.7- to >12-fold) in the DT line ICPA 2039 relative to those of the IDT genotype Asha (Fig. 9). The mean up-regulation of gene expression in the root tip of Asha was observed as 5.32-fold at 15 DAG, 5.69-fold-30DAG, 3.45-fold-10 DAF and 1.94-fold-20DAF. Up-regulation of gene expression in shoot of Asha was observed as 5.68-fold-15DAG, 10.32-fold-30DAG, 11.08-fold-10DAF and 4.68-fold-20DAF. Furthermore, up-regulation of the *CcTFL1* gene (mean expression value is 4.12-fold) in flower tissue at 20 DAF was observed in Asha. In summary, the overall expression pattern of the *CcTFL1* gene in certain tissues was found significantly higher in IDT line Asha when compared to the DT line, ICPA 2039. Expression levels between the IDT and DT accessions in other tissues (root, mature leaf, young leaf and shoot tip) were similar between the DT and IDT accessions, or differed in only specific combinations of tissue and time point (data not shown).

## Discussion

Determinacy is one of the most important and widely studied domesticated traits in flowering plants. In order to obtain early maturing varieties with shorter flowering period, determinacy trait has been selected via domestication process together with photoperiod insensitivity (Repinski et al. 2012). Several studies have been conducted in the past in model plant *Arabidopsis*, pea, soybean, common bean, etc. to identify the genetic mechanism that is responsible for different forms of growth habit (Foucher et al. 2003; Hecht et al. 2005; Kwak et al. 2008; Liu et al. 2010; Tian et al. 2010; Repinski et al. 2012). In some cases it has been proved that determinacy is controlled by single gene, whereas in other studies more than one gene have been found responsible for the transition from vegetative growth to reproductive growth (Tian et al. 2010). In pea, it has been shown recently that the determinate mutant (*det*) is caused by mutations in a homologue of the *Arabidopsis* *TFL1* gene. These mutations are synonymous or non-synonymous substitutions at the junction between an exon and an intron resulting in splicing failure (Foucher et al. 2003). In soybean, the gene responsible for determinacy “*GmTfl1*” has been isolated and found to complement the functions of *TFL1* in *Arabidopsis* (Liu et al. 2010; Tian et al. 2010). Similarly, in common bean, it has been proved that gene “*PvTFL1y*” co-segregated with the determinacy locus “*fin*” (Kwak et al. 2008) and later the same has been validated and found as a functional homolog of *Arabidopsis* *TFL1* gene (Repinski et al. 2012).

The same trait exists in pigeonpea also and the availability of determinate growth habit genotypes having initial vigor and tolerance to drought and water logging is advantageous over indeterminate types for environments with moderate growth (5–6 t ha^−1^) whereas IDT type lines are suitable for environments with high (7–8 t ha^−1^) growth potential (Singh and Oswalt 1992). Some inheritance studies have been conducted earlier in pigeonpea towards understanding the genetics of this important trait (Waldia and Singh 1987; Gupta and Kapoor 1991; Gumber and Singh 1997). We have tried to uncover this mechanism of transition from indeterminate growth habit to determinate growth habit in pigeonpea recently using whole-genome scanning approach using SNPs and DArT assays (Mir et al. 2012b). The present study is in continuation of our earlier efforts towards identification of definite candidates for determinacy in pigeonpea. The identification of candidate gene(s) for determinacy in pigeonpea will allow us to understand the domestication process in pigeonpea and will allow for further, and faster, manipulation of growth habit and flowering time in future breeding efforts.

### Flowering-related genes and sequence diversity

The judicious selection and use of candidate genes during the present study was based on the previous information and validation of their role for determinacy and related traits in *Arabidopsis*, soybean and common bean (Kwak et al. 2008; Tian et al. 2010). Among all the seven genes, *CcTFL1* has been reported as real candidates for the determinacy in these plant species. In pigeonpea, the occurrence of sequence variability in terms of number of SNPs, SNP frequency, nucleotide diversity and number of haplotypes among seven candidate genes strongly indicate the occurrence of different evolutionary constraints. The level of genetic diversity revealed by these gene sequences is in the range of those reported in the literature on crops like *Arabidopsis*, wheat, barley and sunflower (see Giordani et al. 2011). The occurrence of greater sequence diversity in the IDT group than the DT group was likely a manifestation of a domestication or breeding-driven bottleneck experienced by the DT group, which was composed entirely of the cultigen.

Furthermore, nucleotide blast and BlastX results clearly indicated that the correct *TFL1* gene in pigeonpea with same internal structure as that of soybean has been isolated (Tian et al. 2010). Sequence comparison of *TFL1* of all the plant species with pigeonpea *TFL1* (*CcTFL1*) also supported these results as the *CcTFL1* clustered with soybean *TFL1* gene models and common bean *TFL1y* showing maximum similarity (Fig. 2). Similarly, *TFL1* gene sequence of all the DT and IDT lines was found useful in phylogenetic classification/analysis of DT and IDT lines including wild pigeonpea accessions (Fig. 3). In summary, all these results of *CcTFL1* analysis provided great support that the *CcTFL1* of pigeonpea is the same as has been found in other plant species like *Arabidopsis*, soybean and common bean (Kwak et al. 2008; Tian et al. 2010; Repinski et al. 2012).

### Candidate genes for determinacy and linkage analysis

Association analysis through single marker analysis (SMA)/single marker regression showed that this marker based on *TFL1* gene contributes 75 % of phenotypic variation for determinacy in pigeonpea. Further sophisticated analysis using composite interval mapping using QTL Cartographer led to the identification of major QTL on LG09 of pigeonpea genetic linkage map of bi-parental mapping population (ICPA 2039 × ICPR 2447) segregating for determinacy, flowering time and plant height. The major QTL contributes 45–96 % phenotypic variation towards determinacy trait, 45 % towards flowering time and 77 % variation towards plant height and is defined by marker interval *CcTFL1* and CcM0126. Thus these findings clearly indicated that *CcTFL1* controls determinacy in pigeonpea in addition to its role in controlling flowering time and plant height. The other reason for coincidence of several QTLs for these traits could be due to linkage of genes for these traits. The likely control of *TFL1* on more than one trait is also reported in earlier studies in common bean also. For instance, correlation of days to flowering, days to maturity and determinacy were reported in an earlier study in common bean (Tar’an et al. 2002). In addition, it was also found that determinacy causes an early flowering, and there is a positive correlation between earliness and plant height (PH) (Kwak et al. 2008). Mapping of candidate genes with respect to single gene or QTL for growth habit and other related traits provides a test of their possible role in those agronomic traits (Kwak et al. 2008). The isolation and mapping of candidate genes will also test the extent of conserved gene function across multiple crops.

Conversion of SNPs into marker assays revealed that only three candidate genes—*CcAP*, *CcGI* and *CcTFL1* among the seven genes could be either converted into CAPS/dCAPS/PCR-based marker assays. The SNPs in gene *CcAP* and *CcGI* were converted into CAPS and dCAPS assays, respectively, while the SNP in gene *CcTFL1* was converted into user friendly PCR-based marker assay. The sequencing alignment of the *CcTFL1* on 142 pigeonpea germplasm lines (58 DT and 84 IDT lines) led to the discrimination of all the DT lines from the IDT lines with the exception of 4 lines using diagnostic PCR-based SNP assay. The presence of DT allele in four IDT lines could be attributed to some other genes causing variation in growth habit (Ramkumar et al. 2010). Each assay has its own advantages and disadvantages. The CAPS/dCAPS assays require additional steps of long hours with restriction digestion after PCR and sometimes followed by polyacrylamide denaturing gels for fragment separation and silver staining, thus making these markers laborious and costly for regular use in marker-assisted selection (MAS) programs. On the other hand, the PCR-based SNP markers target the functional SNPs by designing PCR primers such that a forward or reverse primer has a specific deoxynucleotide triphosphate (dNTP) at the 3′ end (Collard and Mackill 2008).

The developed marker assays were further directed for genotyping and genetic mapping using either only bi-parental/or bi-parental and wide cross mapping populations. However, only two genes (*CcGI* and *CcTFL1*) could be mapped on the genetic linkage map. The inability to map gene *CcAP* may be due to less number of markers on the map and hence no linkage with any other SSR markers in the genetic map was observed with the CAPS marker. The gene *CcGI* was mapped on linkage group LG02 in the vicinity of two SSR markers (CcM1235 and CcM2241) (Fig. 5). Similarly, candidate gene *CcTFL1* was mapped on the terminal end of LG09 linked by the marker CcM0126 on individual genetic map of ICPA 2039 × ICPR 2447 as well as consensus map of pigeonpea (Fig. 6) developed after merging of several (5–6) genetic maps (Bohra et al. 2012). Candidate genes for determinacy/flowering time have been also mapped in some earlier studies in soybean, pea and common bean (Foucher et al. 2003; Kwak et al. 2008; Tian et al. 2010).

These findings all prove that *CcTFL1* is a likely candidate for determinacy in pigeonpea and the marker based on this gene will prove useful in future marker-assisted breeding programs aiming at pigeonpea improvement by making use of both DT and IDT lines in crossing programs together.

### Comparative genomics analysis and expression profiling of *CcTFL1*

Comparative genomics analysis has been performed to confirm and validate our results that *CcTFL1* is the candidate gene for determinacy in pigeonpea. Comparison with genome sequences of soybean and common bean revealed conservation of eight genes indicating the orthologous nature of *CcTFL1* gene and the high-level of conservation of gene sequence in homologous region across three *Phaseoloid* legumes. In fact, the same genomic region was found to contain *GmTFL1 i*n soybean and *PvTFL1* in common bean (Fig. 8) (Tian et al. 2010; Repinski et al. 2012).

Furthermore, expression profiling of *CcTFL1* supported the results obtained through sequencing and linkage analysis. Overall lower levels of expression of *CcTFL1* were evident in the DT line ICPA 2039 relative to those in the IDT line Asha across multiple tissues and developmental stages (Fig. 9), as it was observed in other legumes such as pea (Foucher et al. 2003) and soybean (Jung et al. 2012). Prior studies have focused on elucidating genes whose expression differs within the same individual, using the different tissue types or between individuals using same tissue (Li et al. 2009; Tian et al. 2010). In the present study gene expression analysis was performed in contrasting genotypes as well as across different developing stage tissues. Further analysis is necessary to elucidate the mechanistic basis for the observed down-regulation of the *CcTFL1* in pigeonpea. In particular it remains to be determined whether the assorting SNP within intron 2 of *CcTFL1* affects transcript stability as observed for regulation of the *RFL* gene in rice (see Prasad et al. 2003) or underlies quantitative control of expression as has been observed in soybean recently (see Ping et al. 2014). In this context, 1,060 bp immediately upstream of the start codon of *CcTFL1* was sequenced for 10 DT and 4 IDT lines (data not shown). Although sequence analysis did not identify polymorphism among DT and IDT lines, the possibility of additional SNP(s) in the cis regions further upstream or in 3′ untranslated regions of *CcTFL1* that may be causal to transition of IDT to DT cannot be excluded. Also of interest is whether the pattern of expression differences between IDT and DT lines may relate to the perennial plant cycle of pigeonpea, which contrasts with the annual habit of other plant species where *CcTFL1* orthologs have been characterized. Nevertheless, our data strongly implicate *CcTFL1* as the likely genetic basis for the evolution of the determinacy trait in cultivated pigeonpea, paving the way for marker-assisted selection for this trait in pigeonpea breeding.

#### **Author contributions**

RKV conceived, designed and coordinated the experiments. RRM, KHB, SS and RKS performed genotyping/experimental setup. RRM, KHB, RKS, RVP and RKV analyzed the data. RKS and KBS performed the field experimentations/selections. RKV, AS and SA contributed reagents/materials/analysis tools. RKV, RRM, KHB, RKS and RVP wrote the paper.

## Electronic supplementary material

Below is the link to the electronic supplementary material.

Supplementary material 1 (XLSX 14 kb)

Supplementary material 2 (DOC 89 kb)
