# Supplementary material for: Candidate gene analysis for determinacy in pigeonpea (Cajanus spp.)
Source: Theor Appl Genet. 2014 Oct 21;127(12):2663–78. doi: 10.1007/s00122-014-2406-8 (PMC4236620; doi:10.1007/s00122-014-2406-8)
Supplement: Supplementary file 2 — Supplementary material 2 (DOC 89 kb) [file 122_2014_2406_MOESM2_ESM.doc]

| **Table S2** List of all nested and degenerate primers used for the amplification of seven candidate genes for determinacy/photoperiod sensitivity | | | |
| --- | --- | --- | --- |
| **Genes** | **Primer name** | **Sequence** | **Reference** |
| ***CcAP1*** | AP1-l1-f | AGCTCATGAGATCTCTGTTC | Kwak et al. 2008 |
|  | AP1-l1-r | AGCGYTCIAGHATCTTCTCC | Kwak et al. 2008 |
|  |  |  |  |
| ***CcFCA*** | FCA-F1 | AAGCAAGCTTTCATTCATCTC | Kwak et al. 2008 |
|  | FCA-R4 | GTAACTCCATATGCCTGG | Kwak et al. 2008 |
|  | FCA-F3 | CAGATGATGCAGCCTTC | Kwak et al. 2008 |
|  | FCA-R6 | CAGTACAGCTATTTAGAACC | Kwak et al. 2008 |
|  |  |  |  |
| ***CcFLD*** | FLD-F1 | TTGGAATATGCAAATGCTGGG | Kwak et al. 2008 |
|  | FLD-R2 | CAGCTTCACCAGCCAC | Kwak et al. 2008 |
|  | FLD-F3 | GCTGGGTGCCTTTCAAATC | Kwak et al. 2008 |
|  | FLD-R4 | CAGCCACCAGCGCAAC | Kwak et al. 2008 |
|  |  |  |  |
| ***CcFKF1*** | FKF1-F1 | GTTGTGKCTGAGATTAG | Kwak et al. 2008 |
|  | FKF1-R2 | GCTATGWCCCCAAG | Kwak et al. 2008 |
|  |  |  |  |
| ***CcGI*** | GI-F1 | GTGATGATGAAGTTGCTCG | Kwak et al. 2008 |
|  | GI-R4 | CATTTGAGCTGTAACTCCAAG | Kwak et al. 2008 |
|  | GI-F3 | GAGAATTTGCACCATTTGGG | Kwak et al. 2008 |
|  |  |  |  |
| ***CcTFL2*** | TFL2-F | TTCTGTCAAGAGGTTCAAGAG | Kwak et al. 2008 |
|  | TFL2-R | TCCACCATCACTTCTGTTCC | Kwak et al. 2008 |
|  |  |  |  |
| ***CcTFL1*** | TFL1-1^2^ | ATGGGGAGAGTGATHGGRGAWG | Kwak et al. 2008 |
|  | TFL1-2^2^ | TCACTAGGRCCWGGAACATCWGG | Kwak et al. 2008 |
|  | TFLC_ph-f | TCTGCAAAGAGAAAAGGAGACA | Kwak et al. 2008 |
|  | TFLC_ph-r | GGTTGAGATTGGTGGAGGAG | Kwak et al. 2008 |
|  | TFL1-3^2^ | GATGTTCCWGGWCCTAGTGAYCC | Kwak et al. 2008 |
|  | TFL1-5^2^ | CTTGCAGCRGTYYTCYCTYTG | Kwak et al. 2008 |
|  | TFLABlong_ph | CTTCTTGTGATGTAAGTGTTTG | Kwak et al. 2008 |
|  | TFLa-f | TGGTTAGTCACTCTCTTACC | Kwak et al. 2008 |
|  | TFLa-r | TCTGTGGATTCCTATCACTG | Kwak et al. 2008 |
|  | GmTfl1-frag1_F | GCACGTCCATGGTACTAGTAGTA | Tian et al. 2010 |
|  | GmTfl1-frag1_R | CAACAAGGAAGAGGAGGAGC | Tian et al. 2010 |
|  | GmTfl1-frag2_F | GGGGCAAAACACACTCGAT | Tian et al. 2010 |
|  | GmTfl1-frag2_R | AGTTCTGTAATGTTTGTTTGAGACT | Tian et al. 2010 |
|  | GmTfl1-frag3_F | GTTTCTCTTAATAACTTAACCTCTT | Tian et al. 2010 |
|  | GmTfl1-frag3_R | TACTACAGAACGTACACAACATCT | Tian et al. 2010 |
|  | Glyma03g35250.1-frag1_F | GATGGAATTAAAAAGAAGTACACTG | Tian et al. 2010 |
|  | Glyma03g35250.1-frag1_R | AAAGAGACTTACTGTGTGAGCTGA | Tian et al. 2010 |
|  | Glyma03g35250.1-frag2_F | TCCTCAGCAGGATTCATCATAAC | Tian et al. 2010 |
|  | Glyma03g35250.1-frag2_R | GAACGGATGATGATGGAGCAT | Tian et al. 2010 |
|  | Glyma03g35250.1-frag3_F | GTTTCTCTTAACAACTCAACCCAA | Tian et al. 2010 |
|  | Glyma03g35250.1-frag3_R | CGGGTCAAACTAGAAAATGATTAAT | Tian et al. 2010 |
|  | PP_transcript asembly _TFL1_F | GGGAAAGAGTTGGTGAGCTATG | Present study |
|  | PP_transcript asembly _TFL1_R | GAAAAACAACCAAGGGAAAGG | Present study |
|  | Gm_Consensus_TFL1_F | ATGGCAAAGATGTGGACAGA | Present study |
|  | Gm_Consensus_TFL1_R | GTTTTTCCCTTTCTCAGTTCA | Present study |
|  | Gm_Consensus_TFL1_F | ATGGCAAAGATGTGGACAGA | Present study |
|  | Gm_Consensus_TFL1_R | AAACAAAAATGCCTTACGTACA | Present study |
|  | Gm_Consensus_TFL1_F | CATCAATGCCTCCTTTTCTC | Present study |
|  | Gm_Consensus_TFL1_R | TTGTGCCTGGAATGTCTGT | Present study |
|  | TFL1_R_Glyma03g35250 | GCATACACACGGGTCAAACTAGAA | Present study |
|  | GmTFL1-1F | AAACGGCAGAAGCTTGACAACT | Present study |
|  | GmTFL1-1R | TTTGGGTCTGGGATGTTTGTG | Present study |
|  | GmTFL1-2F | TGCAAAGGGGAGGGAGGA | Present study |
|  | GmTFL1-2R | GGAGGAGCTAAGGAAGTGAGGA | Present study |
|  | GmTFL1-3F | GGAGGACCCACACACACAGGAT | Present study |
|  | GmTFL1-3R | TTGCCTTTGGGTCTGGGATGT | Present study |
|  | GmTFL1-4F | TGCTTTTTTTGACCTGATCGAG | Present study |
|  | GmTFL1-4R | GGGCTTGGTGTTGACAGTGG | Present study |
|  | GmTFL1-5F | CGAGCTGATGGGAGAGAAAAA | Present study |
|  | GmTFL1-5R | CACCGAAAAAGGGGGACAT | Present study |
|  | GmTFL1-6F | ATCCATGCATCCGGTGTACAG | Present study |
|  | GmTFL1-6R | GCCAGGAACATCAGGGTCAG | Present study |
|  | GmTFL1-7F | ATCCGGTGTACAGCCAATCAA | Present study |
|  | GmTFL1-7R | GGGGTGGGTGTGGTGTGTT | Present study |
|  | GmTFL1-8F | CGATTCCAAGGGCCCCATTCT | Present study |
|  | GmTFL1-8R | GGTGGATGGGATTATGGTGGAA | Present study |
|  | GmTFL1-9F | GCGGAGAGAGAAAGAGAGTGGA | Present study |
|  | GmTFL1-10F | ATCCCAGACCCAAAGGCAATT | Present study |
| **Allele specific for PCR based marker assay of gene CcTFL1** | | | |
|  | TFL1_PCR_CF | GGTACTCATTATACCATCATTTGAG | Present study |
|  | TFL1_PCR_CR | GCATTGAAGTAGACAGCAGC | Present study |
|  | TFL1_PCR_TASF | GGATTCTTTTAACAACTCAACAAAA**T** | Present study |
|  | TFL1_PCR_AASF | GGATTCTTTTAACAACTCAACAAAA**A** | Present study |
|  | TFL1_PCR_TASR | GTACTTTTAAATGATTATCTTAAAAA | Present study |
|  | TFL1_PCR_AASR | GTACTTTTAAATGATTATCTTAAAAT | Present study |
